# Facial Emotion Recognition and Executive Functions in Insomnia Disorder: An Exploratory Study

**DOI:** 10.3389/fpsyg.2020.00502

**Published:** 2020-04-17

**Authors:** Katie Moraes de Almondes, Francisco Wilson Nogueira Holanda Júnior, Maria Emanuela Matos Leonardo, Nelson Torro Alves

**Affiliations:** ^1^Department of Psychology and Postgraduate Program in Psychobiology, Federal University of Rio Grande do Norte, Natal, Brazil; ^2^Postgraduate Program in Psychology, Federal University of RioGrande do Norte, Natal, Brazil; ^3^Department of Psychology, Federal University of Paraíba, João Pessoa, Brazil

**Keywords:** insomnia, facial emotional recognition, sleep deprivation, cognition, executive functions

## Abstract

**Background:**

Clinical and experimental findings suggest that insomnia is associated with changes in emotional processing and impairments in cognitive functioning. In the present study, we investigate the relationship between facial emotion recognition and executive functioning among individuals with insomnia as well as healthy controls.

**Method:**

A total of 11 individuals (mean age 31.3 ± 9.4) diagnosed with insomnia disorder and 15 control participants (mean age 24.8 ± 4.6) took part in the study. Participants responded to a facial emotion recognition task which presented them with static and dynamic stimuli, and were evaluated with regard to cognition, sleep, and mood.

**Results:**

Compared to controls, we found that participants with insomnia performed worse in the recognition of the facial emotion of fear (*p* = 0.001;$\eta_{\text{p}}^{2}$ = 0.549; β = 0.999) and had lower scores in tests of verbal comprehension and perceptual organization (104.00 vs. 115.00, *U* = 135.5; *p* = 0.004; Cohen’s, 2013 *d* = 1.281). We also found a relationship between facial emotion recognition and performance in cognitive tests, such as those related to perceptual organization, cognitive flexibility, and working memory.

**Conclusion:**

Results suggest that participants with insomnia may present some impairment in executive functions as well as in the recognition of facial emotions with negative valences (fear and sadness).

## Introduction

Insomnia disorder (ID) is considered the most prevalent sleep disorder and constitutes a significant burden on health care, causing societal costs (e.g., absenteeism at work, accident risks, a decrease in productivity), functional and cognitive impairment, and increased risk of mental disorders, such as depression (Hillman et al., 2006; Morin et al., 2009; Baglioni et al., 2011; Waters and Bucks, 2011; Suh et al., 2012; Morin et al., 2015; Hertenstein et al., 2018). Some epidemiological studies have shown that 30–50% of the general population complains of some insomnia symptoms (difficulty initiating sleep, difficulty maintaining sleep, waking up too early in the morning). The diagnostic evaluation of insomnia in the general population is uncommon, but a few studies reported 10–15% of individuals meet the criteria for the diagnosis of ID diagnosis (Morin and Benca, 2012; American Psychiatric Association [APA], 2013; American Academy of Sleep Medicine [AASM], 2014; Chaput et al., 2018). According to operational diagnostic criteria, ID is defined as a main complaint of dissatisfaction with sleep quantity or quality, along with difficulty initiating or maintaining sleep, or waking up earlier than expected, not being able to get back to sleep, and followed by a perception of non-restoring sleep (American Psychiatric Association [APA], 2013; American Academy of Sleep Medicine [AASM], 2014). Furthermore, the significant clinical distress and impairment in important areas of daytime functioning must be present.

Clinical and experimental findings have suggested that sleep loss and insomnia is associated with altered emotional processing, such as facial emotion recognition (Tempesta et al., 2018; Zhang et al., 2019). Accurate facial emotion recognition is an important predictor of successful social interactions as it helps to recognize different individuals in their social interactions, to perform accurate interpretations of human faces, and to correctly identify an individual’s current emotional state. Functional deficits in emotion processing have been associated with poor social functioning (Almondes et al., 2016b).

Facial emotion recognition alterations in insomnia patients have recently been presented by a number of studies (Kyle et al., 2014; Crönlein et al., 2016). Some results have shown that ID is associated with a lower accuracy of facial emotion recognition and a reduced rating of emotion intensity for face expressions, as compared to control groups (Kyle et al., 2014; Crönlein et al., 2016). On the other hand, some studies have not found this association between insomnia or sleep disorders and recognition of facial emotions, suggesting that the data are inconclusive (Sheth et al., 2009; Almondes et al., 2016b; Brand et al., 2016, 2019).

In ID, some studies have reported functional and metabolic changes in brain regions associated with emotional processing. For example, Nofzinger et al. (2004), in a functional brain imaging study using ^18^F-fluorodeoxyglucose Positron emission tomography (PET), reported that patients with ID presented, from wakefulness to non-rapid eye movement (NREM) sleep states, relative hypometabolism in brain structures related to emotion and cognitive functioning, including the amygdala, hippocampus, anterior cingulate cortex, and bilateral medial prefrontal cortex (PFC), and in wake-promoting structures including the hypothalamus, thalamus, and ascending reticular activating system. While awake, patients with ID showed smaller decline in relative glucose metabolism in the PFC bilaterally and in other associative areas, and in the thalamus, hypothalamus, and brainstem reticular formation. However, considering the whole brain metabolism, when compared to normal control subjects, ID patients showed greater global metabolism during sleep and while awake (Nofzinger et al., 2004). More pronounced hypoactivation of prefrontal regions and its connections (frontosubcortical and frontoparietal networks) in patients with insomnia was reported by some studies (Altena et al., 2008; Drummond et al., 2013; Stoffers et al., 2014). Overall, it seems that there are interacting neural networks with relative hypometabolism and hypermetabolism, including a general arousal system (ascending reticular formation and hypothalamus), an emotion regulating system (hippocampus, amygdala, and anterior cingulate cortex), and a cognitive system (frontosubcortical and frontoparietal networks). Furthermore, evidence from resting-state functional magnetic resonance imaging (fMRI) indicated that ID appears to alter the functional connectivity in the frontoparietal network (Li et al., 2014).

This data taken together indicates that insomniacs appear to be in a sleep deprivation condition and that this sleep loss seems to be associated with the deterioration of the coding of emotional information, processes of emotional memory consolidation, and the achievement of related cognitive processes of the executive functions (EFs). The EFs are related to emotional and motivational aspects, as well as cost and benefit analysis.

The underlying brain mechanisms of ID are not fully understood, and there is a lack of consensus on how differences in brain activity relate to cognitive and emotional functioning in ID (Kay and Buysse, 2017). However, some neuroimaging studies aggregate relevant findings, especially those showing discrepancies in patterns of brain alterations or activation in emotional contexts. Under sleep deprivation, the amygdala increases responses to negative emotional stimuli, and its connectivity with the PFC is diminished (Motomura et al., 2017). In other lines of evidence with ID, in a sample with a wide range of insomnia severity, restless REM sleep impairs emotional processing in relation to amygdala reactivity (i.e., continued amygdala reactivity). Amygdala reactivity decreased overnight if REM sleep was undisturbed, and individual differences in the reactivity decreasing were proportional to the total duration of REM episodes (Wassing et al., 2019). Patients with ID showed abnormally increased activation in the amygdala from stimuli with insomnia-related content (Baglioni et al., 2014). Also, self-perceived sleep quality modulates the amygdala resting-state functional connectivity in individuals with and without clinical anxiety or depression disorders (Klumpp et al., 2018). Albeit with discrepant findings, most evidence cited so far shows a pattern of amygdala overactivation in the context of emotional stimulus. The PFC top–down connection activity over the limbic region, especially the amygdala and insula, may be disrupted in insomnia and sleep deprivation conditions, leading to emotional dysregulation and alterations, which includes facial expressions. However, it should be noted that brain alterations in ID may be more complex and involve other connections. For example, resting-state fMRI studies show that there are functional disruptions in ID patients, such as disruptions in global and regional topological organization of the brain functional connectome, including the dorsal attention, default mode, and sensory-motor networks (Li et al., 2018).

Evidence also suggests PFC subregions, such as ventromedial and dorsomedial prefrontal areas along with the amygdala and temporal lobe (i.e., inferior and fusiform areas) connections are necessary for a complex executive control of facial recognition and emotional attribution (Vuilleumier and Pourtois, 2007; Heberlein et al., 2008; Todorov, 2012). Beyond emotion association, this relation is important owing to the frontosubcortical and frontoparietal roles in so-called EFs, which refers to an umbrella term denoting a branch of top–down and higher-order cognitive processes involved in the coordination and control of goal-directed behavior, such as inhibitory control, working memory, and cognitive flexibility (Miyake et al., 2000; Chan et al., 2008; Diamond, 2013). Working memory involves holding information temporarily in the mind and mentally working with it to perform a task (Baddeley and Hitch, 1994). Inhibitory control involves being able to control one’s attention, behavior, thoughts, and/or emotions to do what is more appropriate or needed and adaptive (Diamond, 2013). Cognitive flexibility is the ability to change the course of an action or thought and to adjust quickly in favor of the demands and priorities of the environment (Miyake et al., 2000).

Although findings about executive functioning in patients with ID still appear non-consensual, there are findings that indicate that these individuals may present mild to moderate deficits in processes such as working memory, selective attention, problem solving, and inhibitory control (Fernandez-Mendoza et al., 2010; Shekleton et al., 2010; Fortier-Brochu et al., 2012; Suh et al., 2012; Fortier-Brochu and Morin, 2014), which suggests that ID patients can have a reduced ability to engage task-related frontosubcortical and frontoparietal networks. Moreover, there is a discussion that the contradictory literature on the association between insomnia and EFs is essentially due to insomnia phenotypes. Fernandez-Mendoza et al. (2010) and Vgontzas et al. (2013) concluded that only individuals with insomnia with shortened sleep duration, measured objectively through polysomnography, present impairment in EFs.

The current study sought to investigate whether (1) patients with ID would perform differently relative to normal controls on tasks involving facial emotion recognition (happiness, sadness, anger, and fear), whether (2) tasks performance would relate to EF and (3) whether both groups would differ in EF performance. It is hypothesized that patients with ID will present alterations in recognition of facial emotions, that such alterations will be related to EF, and that ID patients will exhibit a lower EF performance when compared to normal controls individuals.

## Materials and Methods

### Participants and Design

A cross-sectional study was conducted. The Insomnia Disorder Group (IDG) comprises participants recruited in the Sleep Clinic AMBSONO of the Federal University of Rio Grande do Norte. Inclusion criteria for participants with ID were as follows: diagnosis of insomnia according to the Diagnostic and Statistical Manual of Mental Disorders – 5th edition (DSM-5) (American Psychiatric Association [APA], 2013), normal or corrected visual acuity, and aged between 20 and 45 years. Exclusion criteria included diagnosis of depression and other psychiatric and neurological disorders, current psychiatric medication use, and dependence on drugs or alcohol.

Normal control group (NCG) participants were recruited through several channels at local universities and public places: online, print, and broadcast media announcements or advertisements for comparative purposes. The inclusion criteria comprised: (a) no sleep disorder; (b) between 20 and 45 years old; (c) normal or corrected visual acuity; (d) not presenting psychiatric disorders; (e) no present dependence on drugs or alcohol.

The total sample (*n* = 26) was composed of 11 participants (four males and seven females; mean age 31.3 ± 9.4 years old) in the IDG and 15 participants (5 male and 10 female; mean age 24.8 ± 4.6 years old) in NCG.

**FIGURE 1:**
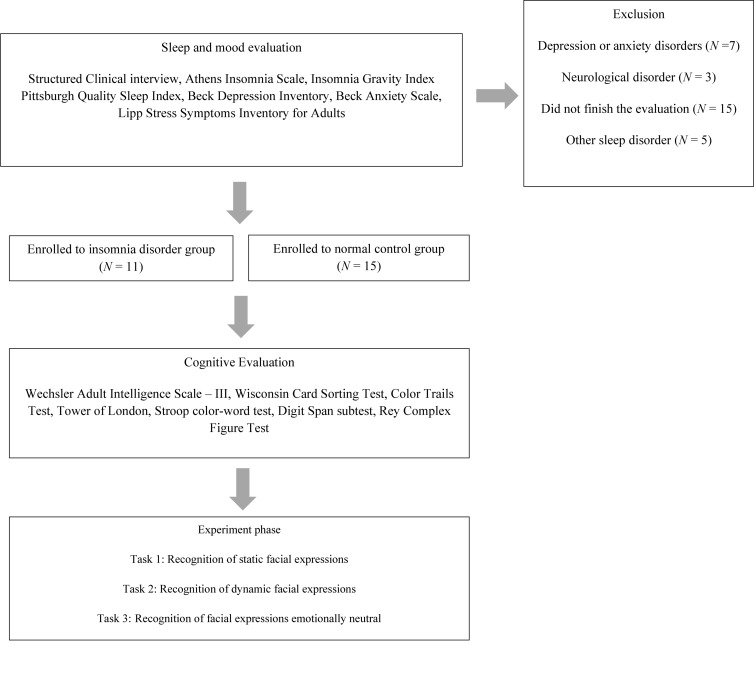
Flowchart of design and procedures of the study.

Participants from both groups were submitted sequentially to a sleep and mood evaluation, a cognitive assessment, and emotional face recognition tasks (Figure 1). This study was approved by the Research Ethics Committee of the Federal University of Rio Grande do Norte (registration number 10843713.2.0000.5537). All participants gave written informed consent for participation. The study was conducted in conformity with the ethical standards proposed in the World Medical Association Declaration of Helsinki.

### Assessments and Procedure

Participants will not receive reimbursement for their participation, since the research was voluntary. The procedures were applied at the laboratory of researchers located at the Federal University of Rio Grande do Norte, between 10 am and 4 pm, in line with participants’ free schedule of activities.

#### Sleep and Mood Evaluation

A structured interview based on clinical diagnostic criteria for insomnia contained in the DSM-5 (American Psychiatric Association [APA], 2013) was conducted. The interview investigated factors such as sleep habits and daytime impairment. The main objective was to answer the inclusion and exclusion criteria, confirming the diagnosis of insomnia participants. This interview was conducted as the first stage of the study (Figure 1).

Participants from both groups completed a number of self-administrated standardized questionnaires commonly used to evaluate sleep, mood, and stress. The severity of insomnia was estimated using the Athens Insomnia Scale (AIS). This is a self-assessment questionnaire consisting of eight items. The total score ranges from 0 (absence of any sleep-related problem) to 24 (the most severe degree of insomnia) (Soldatos et al., 2000). The Insomnia Gravity Index (IGI) was used to assess the severity, nature, and impact of both nighttime and daytime components of insomnia. It is a seven-item self-report questionnaire with a five-point Likert scale to rate each item (e.g., 0 = no problem; 4 = very severe problem), generating a score that ranges from 0 to 28 (Bastien et al., 2001).

The Pittsburgh Quality Sleep Index (PSQI) is a self-report questionnaire that was used to assess sleep quality and quantity over a 1-month period. The global PSQI score represents the sum of the seven subscales (i.e., subjective sleep quality, sleep latency, sleep duration, habitual sleep efficiency, sleep disturbances, use of sleep medication, and daytime dysfunction) and ranges from 0 to 21. Higher scores indicate worse sleep quality (Buysse et al., 1989; Bertolazi et al., 2011). A sleep diary was designed to gather information about an individual’s daily sleep pattern, over a period of 2 weeks. It yielded information about the following measures: sleep fragmentation (SF), total sleep time (TST), sleep efficiency (SE), and restorative sleep perception (RSP) (Carney et al., 2012).

Depressive symptoms were evaluated using the Beck Depression Inventory (BDI). This is a 21-items, self-report rating inventory that assesses the severity of symptoms of depression. The total score ranges from 0 to 63 (Beck et al., 1988b; Cunha, 2001). The Beck Anxiety Scale (BAS) was used to assess the severity of anxiety symptoms. The total score ranges from 0 to 63 (Beck et al., 1988a; Cunha, 2001). The Lipp Stress Symptoms Inventory for Adults (LSSIA) was used to identify and classify stress symptoms (i.e., psychological and physical) among adults according to the four stages of stress: alarm, resistance, quasi-exhaustion, and exhaustion (Lipp, 2000).

### Cognitive Assessment

Cognitive performance was assessed with a series of widely used neuropsychological tests. The Wechsler Adult Intelligence Scale – 3^rd^ edition (WAIS-III) provides a measure of general intellectual function. The four-factor indices were used for cognitive evaluation: Verbal Comprehension, Perceptual Organization, Working Memory, and Processing Speed (Wechsler, 2004). Executive functioning was specifically assessed with the following tests: The Wisconsin Card Sorting Test (WCST) was used to measure the ability to develop abstract concepts (abstract reasoning) and shift between sets (cognitive flexibility and problem solving). The participants have received a set of four reference cards, each differing from the others in terms of three categories: color (red, blue, green, or yellow), shape (triangle, circle, square, or cross), or number (1, 2, 3, or 4). The task is to match the stimuli card to one of four key cards, but the participant is not told how to match the cards, only whether a match is right or wrong. The matching rules change as the test progresses, and participants must adapt their strategy based on feedback from the administrator. The 128-cards version was used. Completed categories, perseverative errors and perseverative responses were used as dependent measures (Heaton et al., 2005).

The Color Trails Test (CTT) was used as a measure of attention (i.e., sustained and divided), processing speed, and cognitive flexibility. It consists of two parts — CTT 1 and CTT 2. For CTT 1, the participant uses a pencil to rapidly connect colored circles numbered 1–25 in sequence. It evaluates perception tracking and sustained attention. For CTT 2, the participant rapidly connects numbered circles in sequence (the numbers 2–25 are presented twice), but alternates between pink and yellow. The CTT 2 evaluates the same functions and introduces a divided attention and cognitive flexibility components because it demands sequential alternations of colors and numbers. The test was scored based on the time, in seconds, to complete the task. An additional score, the interference index, was used for comparison of the participant’s performance on the CTT 1 relative to CTT 2 (D’Elia et al., 2010). Planning capacity and problem solving were evaluated using the Tower of London (ToL). The test comprises three vertical pegs of different lengths and three blocks of different colors (red, blue and green). The blocks must be moved in a certain sequence, peg to peg, from a standard configuration to produce a set pattern (reflected by the examiner’s model) within a specified number of moves. The total score is the sum of obtained points in each sequence problem (Krikorian et al., 1994).

The Stroop color-word test (Victoria Version) is a timed test that evaluates processing speed, attention, and response inhibition. It consists of three separate subtests that involve naming the color of dots (C), of neutral words (W), and of color words printed in incongruent colors (C/W). Each condition contains 24 items. The Color (C) and Word (W) cards were assessed as a first measure of processing speed and sustained attention. The Color/Word (C/W) card was used as a measure of response inhibition. The time needed to finish the cards was recorded (Strauss et al., 2006). The Digit Span Subtest (WAIS-III) is a test that consists of two conditions: digits forward and digits backward, which measures auditory attention and working memory respectively. In both conditions, the examiner reads aloud up to eight (forward) or seven (backward) pairs of random digits, then the participant is asked to repeat the digits in order (Wechsler, 2004). The cognitive assessment also included a measure of visuoconstructive skill and visual episodic memory taken by the Rey Complex Figure Test (RCFT). It comprises a copy trial of the complex figure followed by one more recall trial after 30 min (Rey, 2010).

### Facial Expressions Tasks

**FIGURE 2:**
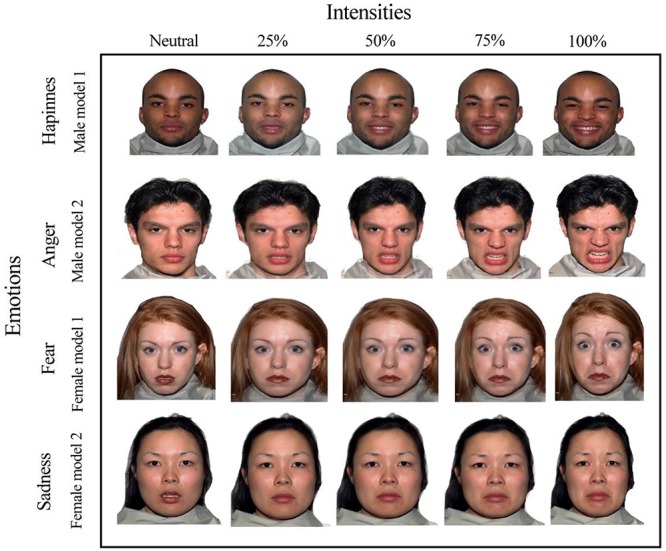
Facial stimuli for the emotions of happiness, sadness, fear, and anger with emotional intensities of 25, 50, 75, 100% and “neutral faces” of two male and female models.

The experimental stimuli were comprised of photographs extracted from the NimStim Face Stimuli Set (Tottenham et al., 2009). The facial expressions of two male and female models showing happiness, anger, fear, sadness, and neutrality were used (Figure 2).

**FIGURE 3:**
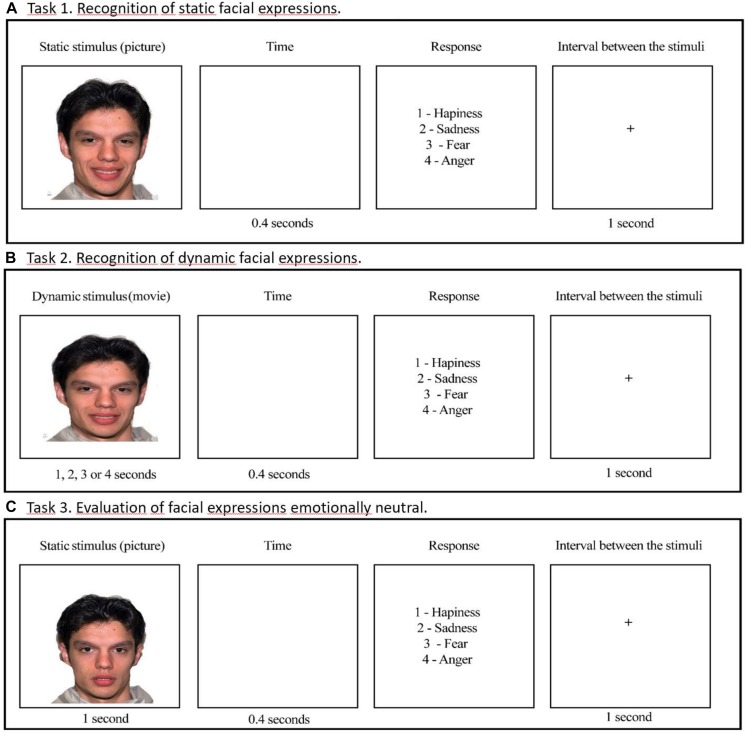
Examples of stimuli presentation in the tasks. **(A)** Task 1: Recognition of static facial expressions. **(B)** Task 2: Recognition of dynamic facial expressions. **(C)** Task 3: Evaluation of facial expressions emotionally neutral.

Three tasks to evaluate facial emotion recognition were designed as follows: recognition of static facial expressions (Task 1), recognition of dynamic facial expressions (videos) (Task 2), and emotional attribution to neutral and emotional faces (Task 3) (Figure 3). The software SuperLab 5.0 was used in the stimuli presentation.

In the individual session participants were instructed, for all tasks, to indicate the most appropriate emotional expression for each presented image, using the numeric keypad on their computer. They could observe each picture for the time stipulated by the system. Response options were given on the screen after each stimulus presentation, with a free response time for the participant.

#### Task 1: Accuracy and Response Time in Recognition of Static Facial Expressions

The Software Morpheus 4.0 was used to produce faces varying in emotional intensity, from a neutral face (0%) to full emotional expression (100%). In this task, we used faces with emotional intensities corresponding to 25, 50, 75, and 100%, which were presented for the times of 1, 2, 3, and 4 s, respectively. We maintained such presentation times to static stimuli in order to follow the presentation pattern of the dynamic stimuli as described in Task 2. In each session, 64 stimuli were presented, composed of 4 expressions (fear, anger, sadness, and happiness) vs. 4 intensities (25, 50, 75, and 100%) vs. 4 models (two males and two females). After the stimulus presentation, participants had to press the corresponding option to the emotion on the keyboard.

#### Task 2: Accuracy and Response Time in Recognition of Dynamic Facial Expressions

The Software Adobe Premiere Pro CS3 was used to produce dynamic facial expressions. The process of creating videos of facial expressions involves the ordering of photos (morphings) with a time criterion of transition from one image to other. Videos began with 1% and increased progressively by steps of 1% to the values of 25, 50, 75, and 100% of emotional intensity. We used the rate of 25 frames per second (FPS). Therefore, dynamic stimuli with 25, 50, 75, and 100% of emotional intensity lasted 1, 2, 3, and 4 s, respectively. A screen with emotional labels was presented after each dynamic stimulus, and the participant had free time to respond by pressing the corresponding key to the emotion on the keyboard.

#### Task 3: Emotional Attribution to Neutral Faces

In this task, we used the same 64 static faces presented in Task 1, with addition to 8 neutral faces, totaling 72 stimuli. We set the presentation time to 1 s for all stimuli in order to increase the difficulty level of the emotional identification task. After observing the facial expressions, including ‘neutral faces,’ the participant should indicate the most appropriate emotional expression. There was not a ‘neutral face’ in the alternative responses and it was not mentioned to the participants that neutral faces would also be presented. Therefore, as they were to choose the emotion corresponding to each face presented, they were forced to assign an emotion to the neutral faces.

### Statistical Analysis

The software SPSS (Statistical Package for the Social Sciences) 21.0 was used for data analysis. In all statistical tests, we assigned the alfa of 0.05 (5%) as the statistical significance criterion. The Shapiro–Wilk (SW) test was used to verify normality. For demographic and sleep variables, the SW test indicated that data does not follow a normal distribution. For this reason, we used the Mann–Whitney test (U) and Chi-square test (X^2^) to compare IDG and NCG groups with regard to these variables. The relationship among sleep measures, cognition, and recognition were evaluated using the Spearman test (r_s_) in case of a non-parametric distribution and using the Pearson test (r) in case of a normal distribution according to the results obtained from the SW test.

The SW test indicated a normal distribution to other variables investigated in the study: (a) accuracy and response time in facial emotion recognition in Tasks 1 and 2; (b) attribution of emotions to neutral faces in Task 3; (3) accuracy and response time in facial emotion recognition in Task 3; and (4) Cognitive measures. In Tasks 1 and 2, variables were submitted to an ANOVA between-within of repeated measures of model 2 groups (insomniacs and controls) x [2 conditions (static and dynamic) x 4 emotions (happiness, fear, anger, and sadness)]. In Task 3, the variable attribution of emotion was submitted to an ANOVA between-within of repeated measures of model 2 groups (insomniacs and controls) x [4 emotions (happiness, fear, anger, and sadness)]. In Task 3, the variable response time and accuracy in emotional recognition were submitted to an ANOVA between-within of repeated measures of model 2 groups (insomniacs and controls) x [4 emotions (happiness, fear, anger, and sadness x 4 emotional intensities (25, 50, 75, and 100%)]. In the ANOVAs, the variable “groups” was taken as a between-subject factor and the variables “conditions,” “emotions,” and “intensities” were taken as within-subject factors. Statistically significant interactions between factors were analyzed with *post hoc* tests using the Bonferroni’s alfa correction. In the ANOVAs, we also calculated effect size ($\eta_{\text{p}}^{2}$) and observed power (β) for main factors and interactions between them.

## Results

These groups were compared in terms of sociodemographic variables, sleep measures, mood, and cognition (Table 1). Groups did not differ with regard to age (*U* = 53.50; *t* = 1.51; *p* = 0.134; *r* = 0.30) or number of male and female participants [X^2^(1) = 0.26; *p* = 0.598].

**TABLE 1:** Descriptive statistics of demographic data, sleep evaluation, mood and cognitive assessment for Insomnia Disorder Group and Normal Control Group.

Groups	Insomnia Disorder Group (*N* = 11)	Normal Control Group (*N* = 15)	*p*
**Demographic characteristics**	***N* (%)**	***N* (%)**	0.59^1^

**Gender**			
Male	4 (36.4)	5 (33.3)	
Female	7 (63.6)	10 (66.7)	
**Age (years)**	**Mean *(SD)***	**Mean *(SD)***	0.13^2^
	31.3 (9.4)	24.8 (4.6)	
**Education**	***N (%)***	***N (%)***	0.95^2^
Primary School	1 (9.1)	1 (6.7)	
High School	7 (63.6)	10 (66.7)	
College	3 (27.3)	4 (26.7)	
**Sleep and mood evaluation**	**Mdn (IQR)**	**Mdn (IQR)**	
AIS	14.0 (5.0)	5.0 (6.0)	<*0.01^2^*
IGI	16.0 (5.0)	5.0 (7.0)	<*0.01^2^*
PSQI	13.0 (6.0)	6.0 (3.0)	<*0.01^2^*
*Sleep diary measures*	**Mdn (IQR)**	**Mdn (IQR)**	
*Sleep fragmentation*	8.0 (8.0)	1.0 (2.0)	<*0.01^2^*
*Total sleep time* (min)	330.0 (140)	466.0 (63.0)	<*0.01^2^*
*Sleep efficiency* (%)	83.0 (26)	99.0 (4.0)	<*0.01^2^*
*Restorative sleep perception*	***n* (%)**	***n* (%)**	<*0.01^2^*
Rested	1 (9.1)	14 (93.3)	
Tired	7 (63.6)	1 (6.7)	
Very tired	3 (27.3)	0 (0)	
BDI	11.0 (5.97)	6.0 (2.93)	<*0.01^2^*
BAS	17.0 (8.44)	7.0 (4.78)	*0.01^2^*
**LSSIA**	***n* (%)**	***n* (%)**	<*0.01^2^*
No stress	1 (9.1)	10 (66.7)	
Alarm	0 (0)	1 (6.7)	
Resistance	6 (54.5)	4 (26.7)	
Quasi-exhaustion	0 (0)	0 (0)	
Exhaustion	4 (36.4)	0 (0)	
**Cognitive assessment**	**Mdn (IQR)**	**Mdn (IQR)**	
*WAIS*			
Verbal comprehension	118.0 (6.0)	125.0 (12.0)	0.06^2^
Perceptual organization	104.0 (11.0)	115.0 (10.0)	<*0.01^2^*
Working memory	113.0 (12.0)	117.0 (18.0)	0.38^2^
Processing speed	117.0 (22.0)	113.0 (6.0)	0.64^2^
WCST – Perseverative responses	20.0 (24.0)	12.0 (13.0)	0.13^2^
WCST – Perseverative errors	11.0 (19.0)	11.0 (10.0)	0.22^2^
WCST – Completed categories	5.0 (2.0)	6.0 (0.0)	0.07^2^
CTT 1	50.0 (22.0)	50.0 (22.0)	0.64^2^
CTT 2	93.0 (41.0)	89.0 (39.0)	0.95^2^
CTT – Measure interference	1.0 (0.0)	1.0 (0.0)	0.76^2^
ToL	33.0 (2.0)	34.0 (2.0)	0.33^2^
Stroop test (C) – time	14.0 (9.0)	15.0 (7.0)	0.44^2^
Stroop test (W) – time	17.0 (9.0)	16.0 (4.0)	0.76^2^
Stroop test (C/W) – time	26.0 (13.0)	22.0 (5.0)	0.44^2^
Digits span forward	9.0 (3.0)	11.0 (3.0)	0.10^2^
Digits span backward	8.0 (2.0)	8.0 (4.0)	0.14^2^
RCFT – Copy	35.0 (1.0)	36.0 (2.0)	0.19^2^
RCFT – Memory	19.0 (6.0)	24.0 (7.0)	0.06^2^

*Data are presented as a function of Means (M), Medians (Mdn), and Interquatile Range (IQR). ^1^*p*-Value refers to chi-square test and generalized Fisher test. ^2^*p* Refers to Mann–Whitney Test. AIS, Athens Insomnia Scale; IGI, Insomnia Gravity Index; PSQI, Pittsburgh Quality Sleep Index; BDI, Beck Depression Inventory; BAS, Beck Anxiety Scale; LSSIA, Lipp Stress Symptoms Inventory for Adults; WCST, Wisconsin Card Sorting Test; CTT, Color Trails Test; ToL, Tower of London; RCFT, Rey Complex Figure Test. The italicized p-values are significant at *p* < 0.05. Significant p-values are presented in italic.*

Severity and clinical impact of insomnia was statistically higher in IDG when compared to the NCG [Mdn_IDG_ = 16.0, IQR_IDG_ = 5.0 vs. Mdn_NC__G_ = 5.0, IQR_NC__G_ = 6.0; *U* = 3.0; *p* = 0.001). Further, IDG presented worse sleep quality (Mdn_IDG_ = 13.00, IQR_IDG_ = 6.0 vs. Mdn_NCG_ = 6.00, IQR_NCG_ = 3.0; *U* = 9.5; *p* = 0.001) (PSQI > 6). Fourteen-days of sleep diaries confirmed subjective sleep complaints in the IDG. As expected, this group presented greater levels of sleep fragmentation (Mdn_IDG_ = 8.00, IQR_IDG_ = 8.0 vs. Mdn_NCG_ = 1.00, IQR_NCG_ = 2.0; *U* = 14.00; *p* = 0.01), poorer sleep efficiency (Mdn_IDG_ = 83%, IQR_IDG_ = 26.0 vs. Mdn_NCG_ = 99%, IQR_NCG_ = 4.0; *U* = 157.00; *p* < 0.01), fewer hours of sleep (Mdn_IDG_ = 330.00, IQR_IDG_ = 140.0 vs. Mdn_NCG_ = 466.00 min, IQR_NCG_ = 63.0; *U* = 154.5; *p* < 0.01) and less perception of restorative sleep [6.7 (tired) vs. 90.9% (tired and very tired); *U* = 11.50; *p* = 0.01] than the NCG. Taken together these findings validate the composition of the groups.

The mood evaluation revealed that depressive (Mdn_IDG_ = 11.00, IQR_IDG_ = 10.0 vs. Mdn_NCG_ = 6.00, IQR_NCG_ = 6.0; *U* = 9.5; *p* = 0.01), anxiety (Mdn_IDG_ = 17.00, IQR_IDG_ = 8.0 vs. Mdn_NCG_ = 7.00, IQR_NCG_ = 5.0; *U* = 20.5; *p* = 0.001) and stress symptoms (54.5 vs. 26.7%; *U* = 22.0; *p* = 0.01) were statistically significantly more present in the IDG than the NCG, although participants from the IDG did not present mood and anxiety disorders.

Regarding cognitive assessment, the Perceptual Organization Index (WAIS III) (POI) was found to be statistically significantly lower in the IDG (Mdn_IDG_ = 104.00, IQR_IDG_ = 11.0 vs. Mdn_NCG_ = 115.00, IQR_NCG_ = 10,0; *U* = 135.5; *p* = 0.004) than in the NCG. The other cognitive domains evaluated did not show any significant differences (Table 1), suggesting that the NCG had a better performance only in non-verbal reasoning and visuospatial integration, which are processes that compose the POI.

### Tasks 1 and 2: Accuracy in Facial Emotion Recognition

The ANOVA showed a statistically significant effect on the main factors “groups” (*F*_1,24_ = 9,454; *p* = 0.005; $\eta_{\text{p}}^{2}$ = 0.283; β = 0.839), “conditions” (*F*_1,24_ = 20,548; *p* = 0.001; $\eta_{\text{p}}^{2}$ = 0.461; β = 0.991), and “emotions” (*F*_3,22_ = 12,084; *p* = 0.001;$\eta_{\text{p}}^{2}$ = 0.335; β = 0.991), as well as statistically significant interactions between the factors “emotions” and “groups” (*F*_3,22_ = 3.149; *p* = 0,030;$\eta_{\text{p}}^{2}$ = 0.116; β = 0.708), and “conditions” and “emotions” (*F*_3,22_ = 5.362; *p* = 0.006;$\eta_{\text{p}}^{2}$ = 0.183; β = 0.848). We found no statistically significant effects for the interactions between “conditions” x “groups” (*F*_1,24_ = 0.14; *p* = 0.908; $\eta_{\text{p}}^{2}$ = 0.001; β = 0.051), and “groups,” “conditions,” and “emotions” (*F*_3,22_ = 0.609; *p* = 0.564; $\eta_{\text{p}}^{2}$ = 0.025; β = 0.151) (Table 2).

**TABLE 2:** Summary of data analysis carried out with variables related to facial emotion recognition.

**Variable**	**Task**	**Main factors**				**Interactions**			
		**Groups**	**Conditions**	**Emotions**	**Intensities**	**Groups vs. (Conditions or Intensities)**	**Groups vs. Emotions**	**Emotions vs. (Conditions or Intensities)**	**Groups vs. Emotions vs. (Conditions or Intensities)**
Accuracy	1 and 2	*p* = 0.005	*p* = 0.001	*p* = 0.001	not	ns	*p* = 0.030 INT1	*p* = 0.006 INT2	ns
Response time	1 and 2	*p* = 0.100	*p* = 0.001	*p* = 0.014	not	ns	ns	p = 0.017 INT3	ns
Emotional attribution to neutral faces	3	*p* = 0.395	not	*p* = 0.003	not	ns	ns	ns	ns
Accuracy	3	*p* = 0.033	not	*p* = 0.001	*p* = 0.001	ns	*p* = 0.045 INT4	*p* = 0.033 INT5	ns
Response time	3	*p* = 0.006	not	*p* = 0.001	*p* = 0.018	ns	*p* = 0.001 INT6	ns	ns

*Here, we present results for main factors and interactions in the ANOVAs. Interactions were analyzed with *post hoc* tests using Bonferroni’s alpha correction. INT1: Insomnia group presented a lower recognition of fear and sadness compared to control volunteers. INT2: Dynamic faces of happiness were recognized better than static faces of happiness. INT3: In the dynamic condition, more time was needed to evaluate sadness compared to fear and happiness. INT4: Insomnia group had a lower recognition of fear compared to control group. INT5: Differences between intensities were found more frequently for happiness than for other emotions. INT6: Insomnia group needed more time to recognize fear and sadness compared to the control group. not: factor not included in the ANOVA model. ns: non-statistically significant.*

**FIGURE 4:**
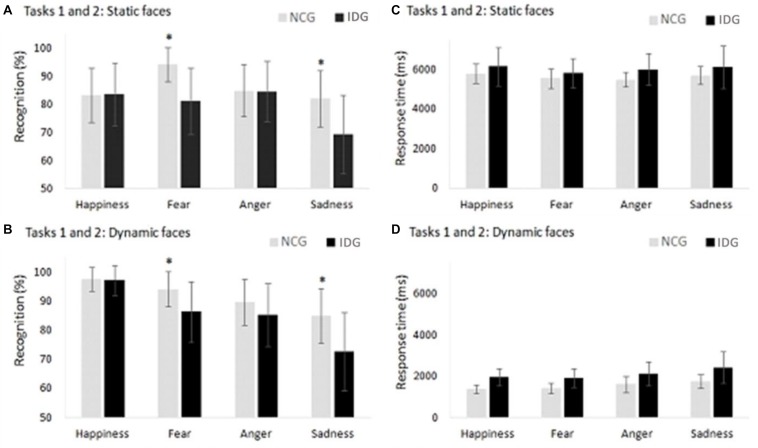
**(A,B)** Control Group (NCG) performed better in the recognition of facial emotions of fear and sadness compared to the Insomnia Disorder Group (IDG). **(C,D)** We found no statistically significant differences between groups with regard to response times in facial emotion recognition in the static and dynamic conditions. Data are presented as a function of means and standard errors. Statistically significant interactions between factors were analyzed with *post hoc* tests using the Bonferroni’s alfa correction (*p < 0.05).

The analysis of the interaction between “emotions” and “groups,” carried out with the Bonferroni’s correction (Table 2), showed that individuals with insomnia presented a lower recognition of facial expressions of fear (*p* = 0.001; $\eta_{\text{p}}^{2}$ = 0.549; β = 0.999) and sadness (*p* = 0.026; $\eta_{\text{p}}^{2}$ = 0.191; β = 0.627) in comparison to control volunteers. Both groups had a similar performance in the identification of happiness (*p* = 0.972; $\eta_{\text{p}}^{2}$ = 0.001; β = 0.050) and anger (*p* = 0.540; $\eta_{\text{p}}^{2}$ = 0.191; β = 0.627) (Figure 4). For the interaction between “conditions” and “emotions,” a *post hoc* test indicated an advantage in recognizing dynamic, compared to static, expressions of happiness in both groups of participants (*p* = 0.001; $\eta_{\text{p}}^{2}$ = 0.628; β = 1.000). For other emotions, there were no differences between the dynamic and static conditions. The analysis of the main factor “emotion” showed a lower recognition for sadness compared to happiness (*p* = 0.001; $\eta_{\text{p}}^{2}$ = 0.588; β = 0.995) and fear (*p* = 0.001; $\eta_{\text{p}}^{2}$ = 0.588; β = 0.995) (Table 2).

### Tasks 1 and 2: Response Time in Facial Emotion Recognition

The ANOVA showed statistically significant effects on the main factors “conditions” (*F*_1,24_ = 756.790; *p* = 0.001; $\eta_{\text{p}}^{2}$ = 0.969; β = 1.000) and “emotions” (*F*_3,22_ = 4.245; *p* = 0.014; $\eta_{\text{p}}^{2}$ = 0.150; β = 0.779) (Table 2). Groups of participants (IDG and NGC) presented similar response times in evaluation of facial emotions (*F*_1,24_ = 2.928; *p* = 0.100; $\eta_{\text{p}}^{2}$ = 0.109; β = 0.376).

We also found an interaction between the factors “conditions” and “emotions” (*F*_3,22_ = 4.211; *p* = 0.017; $\eta_{\text{p}}^{2}$ = 0.150; β = 0.779). *Post hoc* analysis showed no differences between emotions in the static condition. However, in the dynamic condition, more time was needed to evaluate the face of sadness compared to the faces of fear (*p* = 0.013) and happiness (*p* = 0.003) (Figure 4). Other interactions in ANOVA were not significant: “conditions” x “groups” (*F*_3,22_ = 0.296; *p* = 0.591;$\eta_{\text{p}}^{2}$ = 0.012; β = 0.082); “emotions” x “groups” (*F*_3,22_ = 0.270; *p* = 0.806; $\eta_{\text{p}}^{2}$ = 0.011; β = 0.094), “conditions” x “emotions” x “groups” (*F*_3,22_ = 0.313; *p* = 0.755; $\eta_{\text{p}}^{2}$ = 0.013; β = 0.99) (Table 2).

### Task 3: Emotional Attribution to Neutral Faces

In Task 3, we used a forced choice procedure, in which participants had to attribute an emotion to both emotional and neutral faces. In data analysis, we carried out separate ANOVAs for the judgment of neutral and emotional faces (Table 2).

#### Response Time in Emotion Recognition in Task 3

**FIGURE 5:**
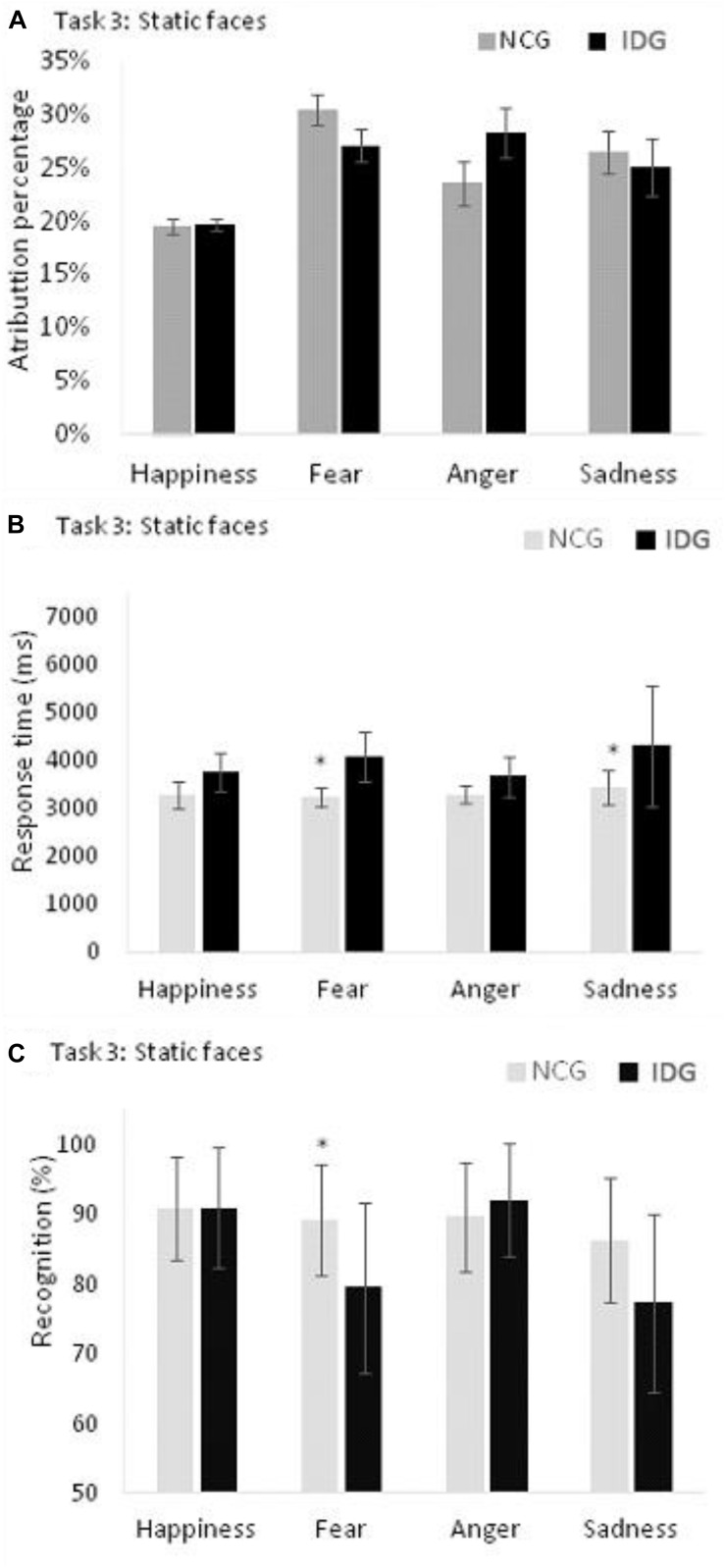
**(A)** Control Group (NCG) and Insomnia Disorder Group (IDG) did not differ in attribution of emotions to neutral faces. **(B)** IDG showed higher response times, when compared to NCG, in recognition of fear and sadness. **(C)** We found statistically significant differences between groups (NCG and IDG) only for fear. Data are presented as a function of means and standard errors. Statistically significant interactions between factors were analyzed with *post hoc* tests using the Bonferroni’s alfa correction (*p < 0.05).

For recognition accuracy, the ANOVA indicated a statistically significant effect on the main factors “groups” (*F*_1_,_24_ = 5.106; *p* = 0.033; $\eta_{\text{p}}^{2}$ = 0.175; β = 0.583), “emotions” (*F*_3_,_22_ = 6,744; *p* = 0.001; $\eta_{\text{p}}^{2}$ = 0.219; β = 0.934), and “intensities” (*F*_3_,_22_ = 65.676; *p* = 0.001; $\eta_{\text{p}}^{2}$ = 0.732; β = 1.000) as well as the interactions “emotions” x “group” (*F*_3_,_22_ = 3.086; *p* = 0.045;$\eta_{\text{p}}^{2}$ = 0.114; β = 0.620) and “intensities” and “emotions” (*F*_9_,_16_ = 2,697; *p* = 0.033;$\eta_{\text{p}}^{2}$ = 0.101; β = 0.743) (Table 2). We found no statistically significant effect for other interactions: “intensities” x “groups” (*F*_3_,_22_ = 0.338; *p* = 0.684; $\eta_{\text{p}}^{2}$ = 0.014; β = 0.097), “intensities” x “emotions” x “groups” (*F*_9_,_16_ = 1.099; *p* = 0.362; $\eta_{\text{p}}^{2}$ = 0.044; β = 0.343). The analysis of the interaction between “emotions” and “groups” showed that participants of IDG had a lower accuracy in the recognition of the expression of fear (*F*_1_,_24_ = 9.423; *p* = 0.005; = 0.283; β = 0.839) compared to the control group. However, groups did not differ with regard to the expression of happiness (*F*_1_,_24_ = 0.007; *p* = 0.933; $\eta_{\text{p}}^{2}$ = 0.001; β = 0.051), anger (*F*_1_,_24_ = 0.643; *p* = 0.430; $\eta_{\text{p}}^{2}$ = 0.026; β = 0.120) and sadness (*F*_1_,_24_ = 3.042; *p* = 0.094;$\eta_{\text{p}}^{2}$ = 0.112; β = 0.388) (Figure 5).

#### Attribution of Emotions to Neutral Faces in Task 3

For the variable attribution of emotions to neutral faces, we found a statistically significant effect on the main factor “emotions” (*F*_3,22_ = 6.643; *p* = 0.003; $\eta_{\text{p}}^{2}$ = 0.217; β = 0.884), but not “groups” (*F*_1,24_ = 0,750; *p* = 0.395; $\eta_{\text{p}}^{2}$ = 0.030; β = 0.132). The interaction between “groups” and “emotions” was not statistically significant (*F*_3,22_ = 1.421; *p* = 0.692; $\eta_{\text{p}}^{2}$ = 0.007; β = 0.067). Bonferroni’s *post hoc* indicated a lower attribution of the emotion of happiness to the neutral faces compared to fear (*p* = 0.001), anger (*p* = 0.010), and sadness (*p* = 0.024) (Figure 5).

For response times, the ANOVA indicated a statistically significant main effect of the variables “emotions” (*F*_3_,_22_ = 11.655; *p* = 0.001; $\eta_{\text{p}}^{2}$ = 0.614; β = 0.998) and “intensities” (*F*_3_,_22_ = 4.041; *p* = 0.018;$\eta_{\text{p}}^{2}$ = 0.360; β = 0.778), “groups” (*F*_1_,_24_ = 9.138; *p* = 0.006; $\eta_{\text{p}}^{2}$ = 0.276; β = 0.826) and a statistically significant interaction between the variables “emotions” and “groups” (*F*_3_,_22_ = 2.231; *p* = 0.001;$\eta_{\text{p}}^{2}$ = 0.542; β = 0.984). Other interactions were not significant: “intensities” and “groups” (*F*_3_,_22_ = 0.201; *p* = 0.895; $\eta_{\text{p}}^{2}$ = 0.027; β = 0.082), “intensities” x “emotions” (*F*_3_,_22_ = 2.151; *p* = 0.087; $\eta_{\text{p}}^{2}$ = 0.547; β = 0.669), and “intensities” x “emotions” x “groups” (*F*_9_,_16_ = 1.220; *p* = 0.349;$\eta_{\text{p}}^{2}$ = 0.407; β = 0.397). The analysis of the interaction between “emotions” and “groups” showed that participants of IDG groups need more time to recognize the expressions of fear (*F*_1_,_24_ = 18.829; *p* = 0.001; $\eta_{\text{p}}^{2}$ = 0.440; β = 0.986) and sadness (*F*_1_,_24_ = 6.030; *p* = 0.022;$\eta_{\text{p}}^{2}$ = 0.201; β = 0.654) compared to the control group. However, groups did not differ with regard to happiness (*F*_1_,_24_ = 4.108; *p* = 0.054; $\eta_{\text{p}}^{2}$ = 0.146; β = 0.494), and anger (*F*_1_,_24_ = 3.825; *p* = 0.062; $\eta_{\text{p}}^{2}$ = 0.137; β = 0.467) (Figure 5 and Table 2).

### Relationships Between Sleep, Cognition, and Recognition of Dynamic and Static Expressions

We carried out two types of correlational analysis: (1) with all participants and (2) for each group (IDG and NCG) (Table 3).

**TABLE 3:** Correlations among measures of sleep, cognition and recognition of dynamic and static expressions.

**Overall (*N* = *26*)**	**Dynamic facial expressions**				**Static facial expressions**			
	**Happiness**	**Fear**	**Anger**	**Sadness**	**Happiness**	**Fear**	**Anger**	**Sadness**
RCFT – Copy	0.079	0.034	–0.296	0.206	−0.448*	0.336	–0.208	–0.041
RCFT – Memory	–0.026	0.049	0.216	0.256	–0.121	0.086	0.196	0.278
WAIS – Verbal comprehension	0.255	0.114	0.077	0.503**	0.110	0.436*	0.277	0.041
WAIS – Perceptual organization	0.288	0.254	0.064	0.305	–0.100	0.439*	–0.040	–0.059
WAIS – Working memory	0.099	–0.012	–0.313	0.292	−0.395*	0.144	–0.121	0.070
WAIS – Processing speed	0.223	–0.260	–0.018	0.023	–0.022	–0.145	–0.097	0.095
Digits span forward	0.078	0.235	–0.278	0.149	–0.235	0.083	–0.166	0.222
Digits span backward	0.706	0.247	0.170	0.469	0.247	0.687	0.419	0.279
Tower of London	0.429*	0.203	0.160	0.285	0.110	0.311	0.025	–0.143
WCST – Generated categories	–0.075	0.217	–0.012	0.186	0.092	0.583**	–0.257	0.126
WCST – Perseverative responses	–0.021	0.034	0.046	–0.281	–0.094	0.506**	0.019	–0.014
WCST – Perseverative errors	–0.045	0.024	0.002	–0.238	–0.127	−0.473*	0.026	–0.005
CTT 1	–0.278	–0.040	0.154	–0.216	0.092	0.001	0.228	−0.411*
CTT 2	−0.406*	0.114	–0.098	–0.033	–0.199	–0.140	0.009	–0.058
CTT – Measure interference	–0.102	0.105	–0.180	0.240	–0.288	–0.191	–0.205	0.312
Stroop test (C) – time	–0.359	–0.252	0.313	–0.268	0.052	–0.136	0.312	–0.099
Stroop test (W) – time	−0.412*	–0.199	0.056	−0.483*	0.036	–0.303	0.041	–0.049
Stroop test (C/W) – time	−0.457*	–0.233	0.029	−0.625**	0.221	−0.438*	0.034	–0.203
**Insomnia disorder group (N = 11)**								
RCFT – Copy	–0.249	–0.563	–0.349	0.032	–0.384	0.372	–0.157	–0.434
RCFT – Memory	0.088	–0.392	0.368	0.119	–0.314	–0.061	0.299	–0.079
WAIS – Verbal comprehension	0.203	0.529	–0.337	0.594	–0.209	0.284	0.061	–0.181
WAIS – Perceptual organization	0.290	0.279	–0.152	0.289	0.169	0.492	–0.078	–0.284
WAIS – Working memory	0.116	–0.220	−0.650*	0.419	−0.604*	0.218	−0.629*	0.201
WAIS – Processing speed	0.493	–0.136	0.344	–0.155	0.067	0.146	0.061	0.161
Digits span forward	0.059	–0.017	–0.488	0.393	–0.506	–0.082	−0.628*	0.444
Digits span backward	–0.030	0.323	−0.634*	0.364	–0.301	–0.218	–0.461	0.423
Tower of London	0.595	0.543	–0.129	0.531	0.140	+0.632*	–0.295	0.148
WCST – Generated categories	–0.092	0.085	–0.208	0.071	–0.054	0.541	–0.473	–0.206
WCST – Perseverative responses	< 0.001	0.005	0.354	–0.506	0.194	−0.671*	0.403	0.046
WCST – Perseverative errors	–0.087	0.034	0.231	–0.318	0.039	−0.607*	0.385	–0.133
CTT 1	–0.467	0.017	–0.134	0.133	–0.040	–0.161	0.284	–0.328
CTT 2	–0.231	–0.348	0.051	0.101	–0.189	–0.295	0.380	0.027
CTT – Measure interference	0.289	–0.372	0.205	0.051	–0.356	–0.212	0.052	0.402
Stroop test (C) – time	–0.556	–0.423	0.387	−0.606*	0.225	–0.455	0.582	–0.259
Stroop test (W) – time	–0.521	–0.443	0.226	−0.684*	0.137	–0.329	0.277	–0.181
Stroop test (C/W) – time	–0.580	–0.403	0.152	−0.688*	0.049	–0.441	0.254	–0.200
**Normal control group (N = 15)**								
RCFT – Copy	0.263	0.017	–0.334	0.121	–0.490	–0.047	–0.333	0.335
RCFT – Memory	–0.145	–0.054	0.017	0.112	–0.045	–0.468	0.137	0.090
WAIS – Verbal comprehension	0.129	–0.386	0.275	0.122	0.645**	0.232	0.600*	–0.380
WAIS – Perceptual organization	0.292	–0.251	0.075	–0.028	–0.056	–0.335	–0.033	–0.035
WAIS – Working memory	–0.070	–0.148	–0.310	–0.037	–0.176	–0.166	–0.158	0.138
WAIS – Processing speed	0.128	–0.445	–0.245	0.540*	–0.103	–0.362	0.034	0.325
Digits span forward	0.102	–0.180	–0.204	–0.41	–0.151	0.104	–0.097	–0.168
Digits span backward	–0.035	–0.002	–0.253	0.211	–0.259	–0.218	–0.196	0.161
Tower of London	0.226	0.072	0.438	–0.126	0.174	–0.68	0.395	–0.501
WCST – Generated categories	–0.314	0.145	0.006	–0.223	0.428	0.103	–0.009	0.015
WCST – Perseverative responses	0.075	0.150	–0.064	0.133	–0.485	–0.084	–0.238	0.260
WCST – Perseverative errors	0.008	0.138	–0.084	0.103	−0.518*	–0.044	–0.278	0.292
CTT 1	–0.186	–0.11	0.280	−0.518*	0.326	0.046	0.199	−0.606*
CTT 2	–0.333	0.505	–0.102	–0.046	–0.102	–0.067	–0.184	–0.114
CTT – Measure interference	–0.283	0.248	–0.513	0.411	–0.319	–0.167	–0.511	–0.440
Stroop test (C) – time	–0.050	–0.009	0.366	0.172	–0.096	–0.345	0.209	0.119
Stroop test (W) – time	–0.025	0.278	0.168	–0.006	–0.018	–0.233	0.041	0.284
Stroop test (C/W) – time	–0.180	0.277	0.009	–0.085	0.416	0.068	0.150	0.099
**Whole group (N = 26)**								
SF	0.134	–0.252	–0.353	–0.024	0.088	–0.372	–0.023	–0.111
SE	–0.054	0.175	–0.084	0.312	–0.338	0.402*	–0.125	0.203
TST	0.208	0.135	0.203	0.568**	–0.129	0.586**	0.077	0.379
**Insomnia disorder group (N = 11)**								
SF	0.319	0.228	–0.421	0.552	0.151	0.264	–0.233	0.248
SE	–0.405	–0.230	–0.478	–0.035	−0.803**	–0.310	–0.371	–0.087
TST	0.115	–0.599	0.074	0.336	–0.527	0.290	–0.019	–0.059
**Normal control group (N = 15)**								
SF	0.423	–0.125	0.232	0.238	0.294	0.322	0.326	0.236
SE	0.191	–0.266	0.091	0.050	0.351	–0.230	0.143	0.172
TST	0.333	–0.402	0.143	0.567*	0.342	–0.169	0.282	0.373

* *p-*Values refers to *p* < 0.05; ** *p* values refer to *p* < 0.01; SF, sleep fragmentation; TST, total sleep time; SE, sleep efficiency; WCST, Wisconsin Card Sorting Test; CTT, Color Trails Test; RCFT, Rey Complex Figure Test.

For correlations with all participants, we found that recognition of the static expression of fear was statistically significantly interrelated to the WCST measures: generated categories (*r* = 0.583; *p* < 0.01), perseverative responses (*r* = −0.506; *p* < 0.01), perseverative errors (*r* = −0.473; *p* = 0.01). Furthermore, the same expression was also associated with the Perceptual Organization – WAIS III (*r* = 0.439; *p* = 0.02), Verbal Comprehension Index – WAIS III (*r* = 0.436; *p* = 0.02), response time from the Stroop Test C/W (*r* = −0.438; *p* = 0.02), SE (*r* = 0.402; *p* = 0.04) and TST (*r* = 0.586; *p* < 0.01).

Accuracy of recognition of the dynamic sadness expression was negatively associated with response time from the Stroop Test W (*r* = −0.483; *p* = 0.01), C/W (*r* = −0.625; *p* < 0.01) and positively associated to the Verbal Comprehension Index – WAIS III (*r* = 0.503; *p* < 0.01). Additionally, recognition of the static expression of sadness was associated with CTT 1 (*r* = −0.411; *p* = 0.03). We found a statistically significant correlation between recognition of sadness and TST (*r* = 0.586; *p* < 0.01). Finally, the recognition of happiness was correlated to the performance in the tests: RFCT – copy (*r* = −0.448; *p* = 0.02), Working Memory Index (WMI) – WAIS III (*r* = −0.395; *p* = 0.04), ToL (*r* = 0.429; *p* = 0.01); CTT 2 (*r* = −0.406; *p* = 0.03), response time from the Stroop Test W (*r* = −0.412; *p* = 0.03) and C/W (*r* = −0.457; *p* = 0.01).

**FIGURE 6:**
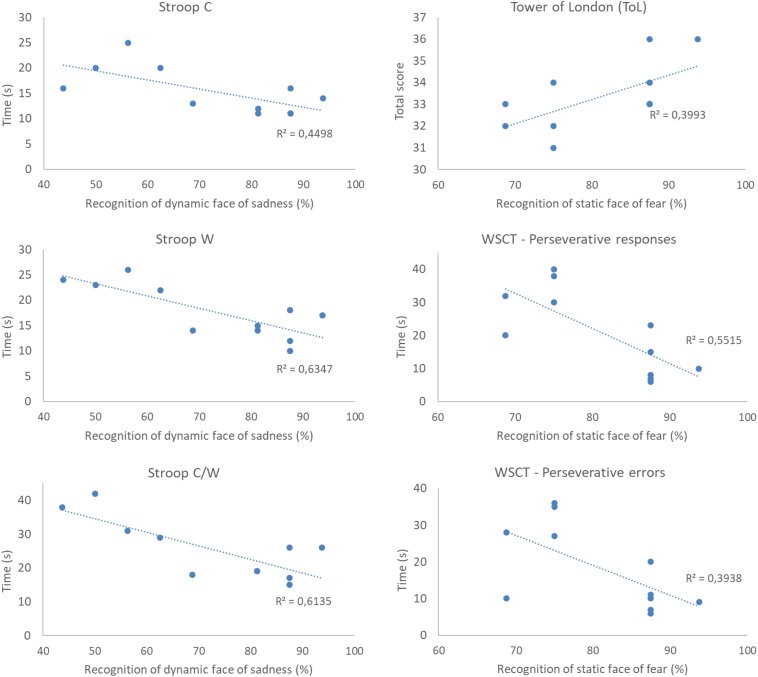
Example of data plotting of correlations between cognitive measures and recognition of the expressions of sadness and fear in participants with insomnia disorder (IDG group). All correlations were significant at the level of 5% (*p* < 0.05). r_s_^2^ represents effect sizes of correlations. As a general result, we see that the performance in the cognitive measures is positively correlated to facial emotion recognition.

For the IDG, there was a statistically significant negative association between the recognition of the static expressions of anger and happiness and the working memory measures – WAIS III (*r*_s_ = −0.629; *p* = 0.03; *r*_s_ = −0.604; *p* = 0.03), and between the dynamic expression of anger, the digits span backward (*r*_s_ = −0.634; *p* = 0.03) and working memory measures – WAIS III (*r*_s_ = −0.650; *p* = 0.03). Recognition of the dynamic sadness expression was negatively associated with response time in the Stroop Test C (*r*_s_ = −0.606; *p* = 0.04), W (*r*_s_ = −0.684; *p* = 0.02), and C/W (*r*_s_ = −0.688; *p* = 0.01). Lastly, perseverative and response errors from WCST were negatively associated with recognition of the static fear expression (*r*_s_ = −0.671; *p* = 0.02; *r*_s_ = −0.607; *p* = 0.04). We also found a positive correlation between the recognition of the static face of fear and ToL scores (*r*_s_ = 0.632; *p* = 0.03). Examples of data plotting of the correlations between cognitive measures and facial emotion recognition are presented in Figure 6.

For the NCG, the accuracy of the recognition of static happiness and anger expressions were positively associated with the Verbal Comprehension Index – WAIS – III (*r*_s_ = 0.645; *p* < 0.01; *r*_s_ = 0.600; *p* = 0.01). The recognition of static and dynamic sadness expressions was identified to have a statistically significant negative association with CTT 1 (*r*_s_ = −0.606; *p* = 0.01; *r*_s_ = −0.528; *p* = 0.04), and recognition of the dynamic face of sadness was positively associated with Processing Speed (WAIS III) (*r*_s_ = 0.540; *p* = 0.03), which suggests that a better accuracy in recognition of these emotions is related to the ability of processing speed and sustained attention in NCG. Additionally, perseverative errors in WCST were negatively related to accuracy of the static happiness expression (*r*_s_ = −0.518; *p* = 0.04). For sleep measures, TST was positively associated with the recognition of the static expression of fear (*r*_s_ = 0.567; *p* = 0.02), which indicated that a high amount of sleep time is related to better accuracy in the recognition of fear.

## Discussion

The present study investigated whether patients with ID had impairments in the ability to recognize facial emotion as well as their relationship to executive functioning. Findings suggest that patients with insomnia have a lower recognition of the facial emotions of fear and sadness, and lower perceptual organization. Insomniacs with impairment in facial emotion recognition had a lower performance in neuropsychological tests related to inhibitory control, planning capacity, problem solving, and cognitive flexibility.

Insomniac individuals reported poor sleep quality, poor sleep efficiency, sleep fragmentation, and perception of non-restoring sleep through their 14-day filled sleep diaries. These findings are consistent with the definition and classification of ID based on subjective complaints (The International Classification of Sleep Disorders – ICSD-3). Groups were balanced with regard to age and sex. The research questions formulated are now discussed.

### Association Between Facial Emotion Recognition and Subjective Symptoms of Insomnia

Overall, the data showed that insomnia may affect the reaction time and accuracy of facial recognition of fear and sadness in static and dynamic conditions. The IDG showed more incorrect responses and needed more time to recognize facial emotions when compared to control individuals. These data are compatible with studies which show that a shorter sleep duration, in the case of insomnia patients, is associated with a lower performance in facial emotion recognition (Cote et al., 2014; Motomura et al., 2014; Almondes et al., 2016b).

Kyle et al. (2014) analyzed emotional categorization and intensity ratings related to the emotions of anger, fear, happiness and sadness. They found that groups did not differ on emotion categorization (i.e., matching the facial expression to the emotion category), but patients with insomnia tended to rate facial expressions of fear and sadness as less emotionally intense than healthy control individuals. Therefore, their findings indicate that people with insomnia may present changes in the evaluation of facial emotions of sadness and fear, in a partial agreement with our results. Furthermore, Maccari et al. (2014) investigated the impact of reduced vigilance due to moderate sleep deprivation on the ability to recognize emotional expressions of faces and the emotional content of words, and they found that positive faces were more resistant than negative faces to the detrimental effect of sleep deprivation. However, there are divergent results about the specific emotions affected by sleep loss. Crönlein et al. (2016) demonstrated that insomnia patients performed worse in recognition of facial emotions of happiness and sadness compared to controls. Killgore et al. (2017) showed that one night of sleep deprivation affects the facial recognition of happiness and sadness. Van der Helm et al. (2010) found that sleep deprivation selectively impairs detection of angry and happy facial emotions. Cote et al. (2014) investigated the impact of sleep deprivation on neural responses to facial emotions as well as on the accuracy and speed of categorization of faces, and found that sleep deprivation preferentially impacted the processing of sadness. Differently, Holding et al. (2017) found that sleep deprivation had no influence on emotional recognition. More recently, Sack et al. (2019) presented videos clips of female senders communicating the emotions of anger, fear, disgust, and sadness to their romantic partner. They found that sleep-deprived participants performed better at recognizing emotion in the videos with a longer presentation time (8–10 s), compared to controls. Considering this literature, it is important to highlight that there are few studies analyzing the association between insomnia and facial emotion recognition.

It is not clear why studies differ with regard to the affected emotions, including our findings. One of the hypotheses may be related to the modality chosen to present the stimulus. In our study, individuals with insomnia and controls had to recognize facial emotions in both static and dynamic conditions. Static conditions may produce more accuracy when used in experiments evaluating the recognition (identity) of unfamiliar faces (Roark et al., 2003). However, dynamic conditions can reflect real-life conditions more deeply. It is important to note that we do not use real dynamic facial expressions as stimuli, here defined as the video recording of a facial expression occurring in an individual in real time. Real dynamic expressions can be expected to have more ecological validity compared to “morphed” dynamic expressions. However, in the literature, we found similar results in studies containing both types of stimuli, with an advantage among the recognition of dynamic expressions compared to static ones (for a review, see Alves, 2013). In the case of our study, the use of dynamic stimuli composed of morphed faces was important to control the exposition time and emotional intensity (25, 50, 75, and 100%) of each stimulus.

Hoffmann et al. (2013) showed that only two emotions (fear and surprise) were easily recognized in dynamic stimuli. Although neuroimaging studies have shown that static and dynamic facial stimuli activate the fusiform gyrus, there are sub-regions close to this region that respond separately to static and dynamic stimuli (Kawasaki et al., 2012). In our study, we found that fear and sadness expressions were judged less accurately by patients with insomnia in both dynamic and static conditions, evidencing the association between insomnia and the recognition of those particular facial emotions.

One of the main findings in literature concerning sleep deprivation and insomnia is the increase of negative moods (anger, depression, fear, and fatigue), which can be associated with depression and anxiety in adults and older adults (Babson et al., 2010; Baglioni et al., 2010; Paterson et al., 2011; Almondes et al., 2016a). For dynamic expressions, we found a positive correlation between the recognition of sadness and TST (Total Sleep Time) indicating that the lower recognition of sadness was associated with less hours of sleep. For static expressions, we found positives correlations between the recognition of fear and the measurement of TST (Total Sleep Time) and SE (sleep efficiency). Kyle et al. (2014) and Holding et al. (2017) did not observe significant associations between markers of sleep deprivation (parameters of sleep diary) and specific emotions. However, Visted et al. (2018) showed in their systematic review and meta-analysis that individuals with a history of depressive episodes (current or remission) reported more difficulties in emotional regulation.

Although insomniac individuals were psychologically healthy, they presented indications of anxiety, stress, and depression. This data may reflect the daytime consequences of perceived sleep loss. Besides, insomnia increases the risk of psychopathology (Hertenstein et al., 2018). Whether impairment in the processing of emotions is due to psychological distress mediated by the fatigue of insomnia, or whether chronic sleep disorder has a direct impact on the processing of emotions, is a field of research for the future.

Threat-related signals and facial expressions of fear are processed mainly by the amygdala structure (Adolphs, 2008; Méndez-Bértolo et al., 2016). Greater activation in the amygdala to threat-related signals and fear expressions has been shown in individuals with chronic insomnia and in sleep deprived subjects, while prefrontal networks exhibit hypoactivation (Huang et al., 2012; Motomura et al., 2013; Baglioni et al., 2014). Maybe the frontal-amygdala route could be disrupted in insomnia, affecting emotional regulation and functioning, which are necessary for recognition of the facial emotion of fear.

### Executive Functions Performance in Insomnia Individuals

There were no statistically significant differences between groups in almost all measures of EFs, with the exception of the Perceptual Organization Index (POI), in which the IDG had a lower performance compared to the NCG. POI is a multi-process measure of non-verbal fluid reasoning, visual perception and integration, and visual-spatial problem-solving. Fluid reasoning and problem-solving are considered more complex components of EFs (Diamond, 2013), and their visual modality (e.g., non-verbal fluid reasoning and visual-spatial problem-solving) relies mainly on frontoparietal networks (Watson and Chatterjee, 2012). Recent investigation has pointed to a disrupted connectivity between the superior frontal gyrus and the superior parietal lobe in patients with insomnia (Li et al., 2014), which would explain the current findings from the IDG.

This data indicates that insomniac individuals can present visual alterations in perception. Chee et al. (2008), Chuah and Chee (2008), and Chee and Tan (2010) have shown that during sleep deprivation there is a decline in visual sensory processing, which could be due to an attenuation of a normal top–down attention biasing system that supports the recognition of familiar faces through the evaluation of stimuli and information processing. Zhang et al. (2019) showed that the impaired perception of facial expressions after sleep loss is associated with a diminishment in the visual attention control and emotional functioning.

### Association Between Executive Functions and Facial Emotion Recognition in Insomnia

Taken together, correlation analysis indicates that individuals with insomnia who had lower recognition of fear presented a worse performance in inhibitory control, planning ability, problem solving, and cognitive flexibility. By this turn, individuals with insomnia who had a lower recognition of sadness presented impairment in inhibitory control.

We found that insomniacs with low recognition of static fear expressions had less ability to plan and solve problems, and more perseverative responses and errors (measured by the Wisconsin Card Sorting Test – the EF test). This result indicates that a low recognition of facial emotions may be associated with a difficulty in identifying the most appropriate strategies in response to a situation of choice or difficulties with problem solving which requires flexibility of reasoning. Facial emotion recognition requires cognitive ability, because there are multiple face details to be identified and differentiated (Calcutt et al., 2017). In addition, the occurrence of a large number of persevering errors can be considered an important behavioral marker for PFC-related dysfunctions (Strauss et al., 2006). The insomniacs also presented non-perseverative errors (errors response), which suggests the adoption of an incorrect classification logic when performing the test. Such performance suggests a state of inattention characterized by a difficulty in focusing attention on the execution of a task.

Executive functions, in general, are superior cognitive processes that allow the maintenance of proper mental functioning to achieve a future goal, being partly responsible for the ability to initiate actions, plan and predict ways of solving problems, anticipate consequences and modify strategies flexibly (Lezak et al., 2004). These functions allow the individual to perform, independently and autonomously, activities aimed at a specific goal which involve complex processes and behaviors. These actions depend on the integrity of various cognitive, emotional, motivational, and volitional processes, which are closely associated with the functioning of the frontal lobes.

Another hypothesis to explain this data is that there is an interaction between PFC subregions, such as ventromedial and dorsomedial prefrontal areas with the amygdala and temporal lobe connections required for complex emotional control and allocation, as well as an adequate functioning of complex order processes such as EFs (Vuilleumier and Pourtois, 2007; Heberlein et al., 2008; Todorov, 2012). Thus, insomnia, a state of sleep deprivation in relation to hypermetabolism relationship at night and hypometabolism during wakefulness (Nofzinger et al., 2004), would lead to an impact on the emotional regulation system as well as in the cognitive processing system.

We found a negative correlation between the recognition of dynamic expressions of sadness and the performance in the Stroop Test, which evaluates sustained attention, processing speed, and inhibition of response. In such a case, participants who had a worse performance in the recognition of the dynamic expressions of sadness also performed worse in the Stroop test (greater response times). Killgore et al. (2017) evaluated the effects of one night of sleep deprivation and one night of subsequent recovery sleep on the ability to identify the six basic emotion categories (happiness, surprise, fear, sadness, disgust, anger). They found that sleep deprivation negatively affected the recognition of facial cues of happiness and sadness, but did not affect the recognition of fear. They argued that survival would be most assured if an individual was able to sustain accurate or enhanced recognition of cues reflecting potential danger (e.g., the face expressing anger, fear, surprise, or disgust), and that sleep deprivation increases the general tendency to perceive facial expressions as “threatening” in appearance. Similar data was found by Cote et al. (2014), in which sleep deprivation led to greater neural reactivity for threat-related negative emotions and a lower performance in the processing of sad faces. This hypothesis seems to be substantiated by the association found in our study between the recognition of static and dynamic expressions of anger and working memory. The insomniacs recognized this facial emotion that is necessary for survival, regardless of their difficulty with working memory. On the other hand, we found that the insomniacs in our research also had low recognition of the expression of fear, which may have been influenced by the data on depression and anxiety symptoms found in this sample.

The cognitive processes most affected in the insomnia group were related to perceptual organization (non-verbal fluid reasoning, visual-motor integration, and visual-spatial problem-solving) and marginally to verbal comprehension; visuospatial organization; and to the ability of developing abstract concepts and cognitive flexibility. Contradictory findings are reported in the literature regarding the presence of EFs impairments in insomnia (Fernandez-Mendoza et al., 2010; Shekleton et al., 2010, 2014; Vgontzas et al., 2013; Ferreira and Almondes, 2014; Fortier-Brochu and Morin, 2014). It might be explained due the use of neuropsychological tests primarily designed to detect neurological lesions (Lezak et al., 2004). Considering that insomnia is not necessarily correlated to neurological lesions, it would be difficult to detect the cognitive changes in insomnia with such tests. Another discussion for conflicting results is that cognitive processes present different outcomes throughout the day, and neuropsychological tests cannot be applied more than once a day because the tests would facilitate adaptation and learning. Our cognitive evaluation data shows impairments in perceptual organization processes, which are considered components of EFs. We use a battery of extensive tests to measure only components of EFs. In addition, when we analyze data in association with facial emotion recognition, the different cognitive processes that are components of EFs are impaired. Thus, our hypothesis is that there is a connection between emotional and cognitive systems, and in insomnia there would be a decreased functional connectivity between the amygdala and the PFC.

An important piece of data for discussion is that there is evidence that age is a factor in altering the ability to recognize expressions of emotion in the face (Dalgleish, 2004). Older people need less environmental/contextual information to properly recognize facial expressions of emotions, and younger people need contextual details to be able to correctly process expressions. Older individuals have a better ability to distribute attentional resources to facial expressions in relation to contextual information (Leitzke and Pollak, 2016). Other studies have found that older people may have a lower hit rate on face recognition tasks compared to younger people because of age-related cognitive impairment (Leime et al., 2013). Considering the results of cognitive impairment is age-related in the case of older people, in our study, our sample of insomniacs was composed of adult individuals who would not have their results influenced by age, so that impairment in EFs and impairment in facial emotions recognition were associated with insomnia.

### Limitations

To the best of our knowledge, this is the first study that investigates the relationship between EFs and facial emotional recognition in insomnia patients. Despite the novelty of the findings, several limitations do not allow for broad generalizations.

First, we rely on self-reports, which, by its own definition, might be biased. We did not employ objective measures of sleep (e.g., polysomnography and actigraphy) to provide a more robust assessment protocol, or to assess sleep stages and sleep-wake parameters related to emotional processing. On the other hand, we rely on the diagnostic evaluation of insomnia according to the International Classification of Sleep Disorders (ICSD-3).

Moreover, the study was designed for participants to report their subjective symptoms of insomnia, reflecting their real-life conditions. We did not perform sleep deprivation research and did not conduct research on all study variables in a laboratory.

Another limitation concerns the small number of participants. This diminished our capacity to detect more statistically significant interaction effects. A great number of people (100 people) had shown interest in the research, but only 56 individuals agreed to participate. However, due to its voluntary nature and lengthy protocol, it became impossible for many to participate. Despite this, in most ANOVA comparisons of main effects and interactions, we found partials eta squared of large size, varying between 0.20 and 0.80. Considering the present work as an exploratory one, we suggest the need for replication and for caution in the interpretation of the results.

Finally, another limitation of the study is that we did not have information and did not assess whether there was a difference between the groups regarding what time the tests were run.

## Conclusion

Insomnia disorder seems to be associated with a lower performance in the recognition of the facial emotions of fear and sadness. Insomniacs with impairment of facial emotion recognition had a lower performance in neuropsychological tests related to inhibitory control, planning capacity, problem solving, and cognitive flexibility. Furthermore, patients with ID may present alterations in perceptual organization.

## Ethics Statement

This study was approved by the Research Ethics Committee of the Federal University of Rio Grande do Norte (registration number 10843713.2.0000.5537).

## Author Contributions

KA, FJ, ML, and NA: author contribution study design, writing the draft, and integration of the authors’ comments. FJ and ML: data gathering. KA and NA: interpretation of the data and final manuscript.

## Conflict of Interest

The authors declare that the research was conducted in the absence of any commercial or financial relationships that could be construed as a potential conflict of interest.
